# Point of Care Ultrasound (POCUS) Utilization and Barriers by Senior Emergency Medicine and Critical Care Residents at Two Teaching Referral Hospitals, Addis Ababa, Ethiopia

**DOI:** 10.1155/2023/7584670

**Published:** 2023-03-18

**Authors:** Ayalew Zewdie Tadesse, Temesgen Beyene Abicho, Dirijit Mamo Alemu, Anne Aspler

**Affiliations:** ^1^St Paul's Hospital Millennium Medical College, Addis Ababa, Ethiopia; ^2^Addis Ababa University, Addis Ababa, Ethiopia; ^3^University of Toronto, Toronto, Canada

## Abstract

**Background:**

POCUS has become an integral part of the practice of emergency medicine. POCUS is a highly focused, limited, goal-directed exam with the expressed purpose of answering selected questions used at the bedside for critically ill patients who are not stable. We aimed to assess POCUS utilization and barriers by senior-year emergency medicine and critical care residents at two tertiary academic and referral hospitals in Addis Ababa, Ethiopia. *Methodology*. A cross-sectional study was conducted from June 1 to August 30, 2022 in St Paul's Hospital Millennium Medical College and Tikur Anbessa Specialized Hospital using an electronic survey of senior-year (second and third years) emergency medicine and critical care residents. Data were collected using Goggle form, exported to SPSS version 24, and then analyzed.

**Result:**

Seventy-six residents out of 78 (97.4%) responded to the online survey. The mean age was 29.9 years with an SD of 2.87. Fifty-six residents (73.7%) were male and 45 (59.2%) were year 2 residents. Sixty-one (76.3%) had previous POCUS training. Fifty residents (82.0%) received training from the classroom. Twenty-seven residents (35.5%) rated their current level of knowledge as good for sterile transducer techniques, 28 (36.8%) rated fair for their knobology, and 27 (35.5%) rated very good for their transducer selection knowledge. Thirty-two (42.1%) rated very good about their ability to interpret IVC. 26 (34.2%) responded that they had good ability to interpret FAST/EFAST. Forty-nine (64.5%) residents claimed lack of an ultrasound machine followed by 33 (43.4%) lack of organized curriculum were the main barriers to POCUS utilization. Forty-two (55.3%) residents preferred to complete face-to-face teaching, while 33 (43.4%) preferred blended learning both face-to-face and online.

**Conclusion:**

POCUS is performed by the majority of EMCC residents. The most frequent scans performed by residents were FAST, IVC, and lung scans. Lack of ultrasound machine and organized curriculum was the main barrier to US utilization. Availability of equipment, face-to-face training, and having an organized curriculum are recommended by residents to improve their skills in the future.

## 1. Introduction

Point of care ultrasound (POCUS) is one of the most useful imaging tools that can be performed at a patient's bedside in the emergency department for those patients who are critically ill and injured. It can be used for diagnosis, and during resuscitation, it improves accuracy and safety of the procedures [1]. Emergency ultrasound or POCUS is a highly focused, limited, goal-directed exam with the expressed purpose of answering selected questions [2]. POCUS acts as a clinical decision support when it is performed by properly trained physician and does not replace comprehensive imaging where suspected findings are later confirmed with a comprehensive radiographic study [3]. POCUS is currently used at bedside for critically ill patients who are not stable to be transported for comprehensive scanning; procedural guidance, where POCUS helps physicians gain easy access for pericardiocentesis, thoracentesis, central venous line access, and other procedures by facilitating direct visualization in real time; and basic applications such as foreign body localization and selected musculoskeletal imaging [4, 5]. POCUS has become an integral part of the practice of emergency medicine and trauma care. It is routinely used in most emergency medicine residencies and level I trauma centers. It has become part of the advanced trauma life support guideline as a diagnostic tool in trauma patients and is a skill required in emergency medicine training [6]. According to emergency ultrasonography guidelines created by the ACEP in 2001, the seven ultrasound skills of trauma, pregnancy, abdominal aorta, cardiac, biliary, urinary tract, and procedural are included in the scope of practice for EM POCUS. Thoracic, deep vein thrombosis (DVT), ocular, and soft tissue/musculoskeletal were added to this list in 2009 [7, 8].

In Addis Ababa, emergency medicine and critical care residency training program is available at Addis Ababa University/Tikur Anbessa Specialized Hospital (AAU/TASH) and St Paul's Hospital Millennium Medical College (SPHMMC). Residents work at different areas (red, orange, yellow/green) in the emergency department and intensive care unit (ICU). POCUS is integrated in their three year residency curriculum.

There is a lack of literature about POCUS utilization and barriers by emergency medicine and critical care residents in Ethiopia. Hence, we aimed to assess POCUS utilization and barriers by senior-year residents at two tertiary academic and referral hospitals in Addis Ababa, Ethiopia.

## 2. Methods

An institutional-basedcross-sectional study over a period of 3 months from June 1 to August 30, 2022 was conducted from emergency medicine and critical care departments of Addis Ababa University/Tikur Anbessa Specialized Hospital (AAU/TASH) and St Paul's Hospital Millennium Medical College (SPHMMC) located in Addis Ababa, Ethiopia.

TASH Emergency Medicine Department is a pioneer specialty training center in the country, established and started postgraduate residency training in 2010 through collaboration with the University of Toronto and the University of Wisconsin. The program has so far trained nine batches of specialists in emergency and critical care medicine residency programs with nearly 65 graduates who are working all over the country.

SPHMMC is the second biggest teachings and referral hospitals that followed in the footsteps of TASH and established an emergency and critical care medicine department in 2015. Four batches with 37 emergency and critical care medicine specialists graduated.

All senior-year (year 2 and year 3) emergency medicine and critical care residents in TASH and SPHMMC were included in the study. There were 32 senior-year residents at TASH and 46 at SPHHMC. Those residents who were unwilling to participate in the study and not responding to the online survey were excluded. Only 2 residents did not respond to the online survey and were excluded from the study.

Data were collected using pretested and standardized questionnaires. Two data collectors were trained about the study and collected the questionnaire from the residents of both institutions. The questionnaire was sent to the residents via their emails using Google Forms. The questionnaire includes demographics data, level of education, POCUS training, POCUS knowledge questions, POCUS practice questions, barriers for POCUS utilization, and open-ended questions on their recommendation on how to improve POCUS utilization and skill. The primary investigator assessed the completeness of the data.

Data collected using Goggle form were exported to excel, then the data was cleaned, coded, and entered to the software SPSS version 24. A descriptive report was made on the demographics, POCUS knowledge and practice response, and barriers to POCUS utilization. Frequency, percentage, means, and SD were used to describe the result. The result is presented as tables and figure.

## 3. Result

Seventy-six residents out of 78 responded to the online survey with a response rate of 97.4%. The mean age was 29.9 years with a SD of 2.87. Fifty-six residents (73.7%) were male and 45 (59.2%) were year 2 residents (Table 1). Sixty-one (76.3%) had previous POCUS training. Twenty-five (41.0%) were satisfied, while 22 (36.1%) were neutral, 8 (13.1%) dissatisfied, 5 (8.2%) strongly satisfied, and 1 (1.6%) was strongly dissatisfied about their previous training. Fifty (82.0%) residents received training from the classroom, whereas 20 (32.8%) from peers, 18 (29.5%) online, 3 (4.9%) conference, and 1 (1.6%) informally. Twenty-three (37.7%) had more than one source of POCUS training. Residents' response regarding the rate of their current level of knowledge about the basics of POCUS and their ability to interpret different scans is shown in Table 1.

Twenty-seven residents (35.5%) rate their current level of knowledge good for sterile transducer techniques, 28 (36.8%) rated fair for their knobology, 27 (35.5%) rated very well for their transducer selection knowledge, and 40 (52.6%) rated poor for their Spectral Doppler imagining level of expertise. Thirty-two (42.1%) rated very good about their ability to interpret IVC. Twenty-six (34.2%) responded a good ability to interpret FAST/EFAST. Twenty-seven (35.5%) residents reported poor ability to interpret FATE PROTOCOL (focused assessment transthoracic echo) and FASH PROTOCOL (focused assessment with US in HIV pt) (Table 2).

Thirty-seven (48.7%) residents performed more than 100 FAST, lung scans, and IVC scans. Fifty-three (69.7%) residents did not perform any ultrasound-guided nerve block (Table 3).

**Figure 1:**
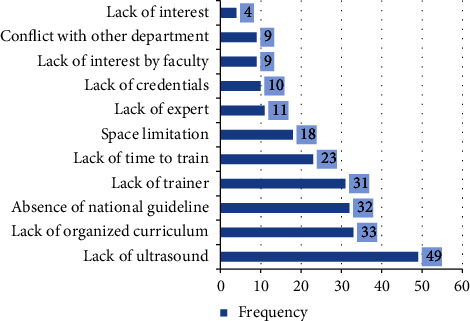
Barriers for POCUS utilization by senior emergency medicine and critical care residents in SPHMMC and AAU/TASH, Addis Ababa, Ethiopia, June 1–August 30, 2022 (*N* = 76).

Forty-nine (64.5%) residents claimed lack of ultrasound followed by 33 (43.4%) lack of organized curriculum were the main barriers for POCUS utilization (Figure 1).

Forty-two (55.3%) residents preferred complete face-to-face teaching, while 33 (43.4%) preferred blended learning both face-to-face and online and only 1 (1.3%) said completely online learning in the future.

For the open-ended question “what recommendation do you have to improve POCUS utilization and skill in the ED 43/76 (56.5%) suggested change in education and training such as face-to-face training, practice, and images discussion during the morning sessions, 22/76 (28.9%) recommend availing of ultrasound machine in the emergency room, 16/76 (21.5%) recommended national protocol and policy development about POCUS use such as accreditation, fellowship program, and standardization.”

A few of the selected comments from the participants are shown as follows:

“Face-to-face bedside POCUS teaching activity and mentoring should be encouraged. Lectures on POCUS are not enough and curriculum and guidelines should be prepared so that everybody can practice based on that. Proper machine video recording (ultrasound saved DICOM) of POCUS should be reviewed in the morning discussion and a case presentation of POCUS should be included in the seminar discussion. Availing internationally credited subscription ultrasonography lecture videos or preparing those if possible will help in self-practice of real-time imaging starting emergency ultrasonography and POCUS fellowship will increase and ease the teaching-learning process in the emergency” ER57.

“I recommend supervision by experts and also to avail ultrasound in every station to practice and also to give high-quality care to our patients because currently ultrasound is considered as 6th sense for physician… and also to prepare modules and guideline to help us the scope and depth of the use” ER63.

“It should be included in the curriculum as one subject matter. Logistic issues should be addressed such as ultrasound and jelly. Training should be given regularly” ER22.

## 4. Discussion

Emergency ultrasonography, also known as POCUS, is a procedure carried out by an emergency physician at the patient's bedside. Its specific objectives enable the doctor to act quickly, discover potentially fatal diagnoses, and speed up the surgical care of emergency patients [2, 9]. It has been a crucial component of EM for more than 20 years, and its use continues to advance [10].

POCUS education is integrated with EMCC curriculum in Ethiopia. Out of 76.3% of residents who had previous POCUS training, 82.0% got training from the classroom. 41% were satisfied with their previous training which showed there should be a way of improving their POCUS education. Starting a POCUS rotation in their ED attachment and evaluating residents based on their performance are some strategies to increase resident satisfaction and skill. Other strategies include practicing at the bedside with consultants, reporting their scans during the morning sessions, and receiving feedback.

From the basics of POCUS, transducer selection knowledge, and *B* mode imaging received the highest percentage of positive responses from residents. In contrast, sterile transducer techniques, *M* mode imaging, color Doppler, image improvement techniques, and ultrasound artifacts received only fair ratings from respondents. However, the majority of respondents said that they lacked knowledge or expertise in spectral Doppler imaging PW and CW Doppler. A higher percentage of residents gave their ability to interpret FAST/EFAST [11, 12] and IVC ratings of very excellent; they gave ECHO, soft tissue scans (cellulitis, abscess), and DVT scans of acceptable; and ONSD of poor. During their residency, most residents did more than 100 EFAST, lung scans, and IVC scans, as well as 5–50 DVT and Echo scans.

The majority of residents conducted less than 5 ultrasound-guided pericardiocentesis and ultrasound-guided central line insertions. Central lines are not available in the hospital and few got their chance of insertion from donations. Most residents performed 5–50 ultrasound-guided paracentesis, thoracentesis, and peripheral intravenous catheterizations. The majority of residents did not conduct arthrocentesis, nerve blocks, or ultrasound-guided abscess drainage. Education through SimLab and practice could help if the cases are low. Based on the suggestions of professional societies, the American College of Graduate Medical Education (ACGME) has made POCUS a necessary component of emergency medicine training. All EM residencies with ACGME accreditation offer POCUS training under consensus recommendations made by the ACEP in 2009, which call for at least 80 hours of focused clinical ultrasonography, 20 hours of didactic ultrasonography education, and accurate completion of 150 independently reviewed ultrasound studies [13]. Future residency education in these settings should thus emphasize the areas in which residents need to improve and build up schedules based on ACGME recommendations with local adjustments.

From the protocols, RUSH, BLUE, and RADiUS were scored as having the fair interpretive ability, while FATE and FASH were classified as having the low interpretive ability. These are ultrasound screening methods to look for extrapulmonary TB (FASH), respiratory distress (BLUE and RADiUS), shock (RUSH), and respiratory with circulatory problems (FATE) [14–18].

Lack of ultrasonography, a lack of organized curriculum, a lack of a national standard, and a lack of training were the top barriers to US usage raised by residents. Lack of ultrasound equipment used to be a significant issue when POCUS first began in the West, but lack of training was also a significant issue in other nations [19–22].

The majority of residents preferred face-to-face POCUS education and recommended it for the future. However, according to a survey conducted at the Hamad General Hospital in Doha, Qatar, flipped classroom (FC) learning was chosen over face-to-face learning for improving POCUS knowledge [23]. Residents recommended availing ultrasonography, developing POCUs policy and protocol, and increasing educational platforms to increase the use and POCUS skill. The creation of a POCUS fellowship program could aid in organizing, directing, and training residents in POCUS skills.

### 4.1. Limitation

The study has several limitations. There was no way to verify if residents had performed these scans because the study asked about their previous years' ultrasonography usage, which leaves room for recall bias by residents. A good documentation form should be created for future use, and data may be verified using the registry. The study did not assess how using ultrasonography will facilitate patient care was its second limitation, which calls for additional research.

## 5. Conclusion

POCUS is performed by the vast majority of EMCC residents. The most frequent scans performed by residents were FAST, IVC, and lung scans. Lack of ultrasonography and organized curriculum were the main barriers to US utilization, and availing ultrasound, face-to-face training, and having an organized curriculum are recommended by residents to improve their skills in the future.

## Figures and Tables

**Table 1:** Sociodemographic characteristics of senior emergency medicine and critical care residents in SPHMMC and AAU/TASH, Addis Ababa, Ethiopia, June 1–August 30, 2022 (*N* = 76).

Variable	Category	Frequency	Percent
Age	<35	70	92.1
	≥35	6	7.9

Sex	Female	20	26.3
	Male	56	73.7

Year of residency	Year 2	45	59.2
	Year 3	31	40.8

Institution	AAU/TASH	33	43.4
	SPHMMC/AABET	43	56.6

Previous POCUS training	Yes	61	80.3
	No	15	19.7

**Table 2:** Current level of knowledge or skills related to POCUS of senior emergency medicine and critical care residents in SPHMMC and AAU/TASH, Addis Ababa, Ethiopia, June 1–August 30, 2022 (*N* = 76).

How would you rate your current level of knowledge or skills in the following domains?	Very poor *n* (%)	Poor *n *(%)	Fair *n *(%)	Good *n *(%)	Very good *n* (%)
Sterile transducer techniques	5 (6.6)	10 (13.2)	28 (36.8)	27 (35.5)	6 (7.9)
Knobology	4 (5.3)	12 (15.8)	28 (36.8)	21 (27.6)	11 (14.5)
Transducer selection	2 (2.6)	1 (1.3)	24 (34.6)	22 (28.9)	27 (35.5)
*B* mode imaging	2 (2.6)	6 (7.9)	22 (28.9)	29 (38.2)	17 (22.4)
*M* mode imaging	4 (5.3)	2 (2.6)	27 (35.5)	22 (28.9)	21 (27.6)
Color Doppler imaging	4 (5.3)	24 (31.6)	28 (36.8)	13 (17.1)	7 (9.2)
Spectral Doppler imaging-pulsed wave	8 (10.5)	39 (51.3)	21 (27.6)	4 (5.3)	4 (5.3)
Spectral Doppler imaging-continuous wave	13 (17.1)	37 (48.7)	21 (27.6)	3 (3.9)	2 (2.6)
Image improvement techniques	4 (5.3)	11 (14.5)	31 (40.8)	19 (25)	11 (14.5)
Ultrasound artifacts	1 (1.3)	18 (23.7)	27 (35.5)	20 (26.3)	10 (13.2)
Ability to interpret FAST/EFAST	4 (5.3)	0 (0.0)	25 (32.9)	21 (27.6)	26 (34.2)
Ability to interpret IVC scan	3 (3.9)	1 (1.3)	23 (30.3)	17 (22.4)	32 (42.1)
Ability to interpret ECHO	3 (3.9)	18 (23.7)	30 (39.5)	23 (30.3)	2 (2.6)
Ability to interpret soft tissue scan (cellulitis, abcess)	5 (6.6)	10 (13.2)	37 (48.7)	20 (26.3)	4 (5.3)
Ability to interpret DVT scan	2 (2.6)	2 (2.6)	33 (42.1)	25 (32.9)	14 (18.4)
Ability to interpret ONSD	6 (7.9)	23 (30.3)	20 (26.3)	18 (23.7)	9 (11.8)
RUSH PROTOCOL (rapid US assessment in shock)	2 (2.6)	5 (6.6)	31 (40.8)	17 (22.4)	21 (27.6)
BLUE PROTOCOL (bedside lung ultrasound in emergency)	2 (2.6)	4 (5.3)	29 (38.2)	22 (28.9)	19 (25.0)
RADiUS PROTOCOL (rapid assessment of dyspnea in ultrasound)	3 (3.9)	14 (18.4)	25 (32.9)	23 (30.3)	11 (14.5)
FATE PROTOCOL (focused asst transthoracic echo)	5 (6.6)	27 (35.5)	23 (30.3)	15 (19.7)	6 (7.9)
FASH PROTOCOL (focused assessment with US in HIV pt)	7 (9.2)	27 (35.5)	23 (30.3)	10 (13.2)	5 (6.6)

**Table 3:** Number of POCUS procedures performed by senior emergency medicine and critical care residents in SPHMMC and AAU/TASH, Addis Ababa, Ethiopia, June 1–August 30, 2022 G.C (*N* = 76).

How many procedures have you performed to date with ultrasound during residency?	None *n* (%)	<5 *n* (%)	5–50 *n* (%)	50–100 *n* (%)	>100 *n* (%)
FAST	0 (0.0)	0 (0.0)	22 (28.9)	17 (22.4)	37 (48.7)
DVT scan	0 (0.0)	8 (10.5)	48 (63.2)	14 (18.4)	6 (7.9)
Lung scan	1 (1.3)	0 (0.0)	20 (26.3)	18 (23.7)	37 (48.7)
ECHO	2 (2.6)	7 (9.2)	46 (60.5)	12 (15.8)	9 (11.8)
IVC scan	0 (0.0)	0 (0.0)	17 (22.4)	22 (28.9)	37 (48.7)
ONSD	11 (14.5)	16 (21.1)	34 (44.7)	11 (14.5)	4 (5.3)
First trimester pregnancy	22 (28.9)	31 (31.0)	18 (23.7)	5 (6.6)	0 (0.0)
US-guided paracenteses	2 (2.6)	22 (28.9)	42 (55.3)	6 (7.9)	4 (5.3)
US-guided central line insertions	15 (19.7)	38 (50.0)	17 (22.4)	5 (6.6)	1 (1.3)
US-guided thoracenteses	1 (1.3)	18 (23.7)	42 (55.3)	11 (14.5)	4 (5.3)
US-guided pericardiocentesis	28 (36.8)	34 (44.7)	13 (17.1)	1 (1.3)	0 (0.0)
US-guided arthrocentesis	33 (43.4)	32 (42.1)	11 (14.5)	0 (0.0)	0 (0.0)
US-guided abcess drainage	36 (47.4)	32 (42.1)	8 (10.5)	0 (0.0)	0 (0.0)
US-guided peripheral intravenous catheterizations	12 (15.8)	24 (31.6)	37 (48.7)	1 (1.3)	2 (2.6)
US-guided nerve block	53 (69.7)	18 (23.7)	5 (6.6)	0 (0.0)	0 (0.0)

## Data Availability

The data used in this study are available from the corresponding author upon request.

## References

[1] Bashir K., Azad A. M., Hereiz A., Bashir M. T., Masood M., Elmoheen A. (2021). Current use, perceived barriers, and learning preference of point of care ultrasound (POCUS) in the emergency medicine in Qatar–A mixed design. *Open Access Emergency Medicine*.

[2] Heller M. B., Mandavia D., Tayal V. S. (2002). Residency training in emergency ultrasound: fulfilling the mandate. *Academic Emergency Medicine*.

[3] Michalke J. A. (2012). An overview of emergency ultrasound in the United States. *World journal of emergency medicine*.

[4] Evans C. H., Cemaj S. (2018). Ultrasound imaging for the surgical intensivist. *Surgical Critical Care Therapy*.

[5] Smallwood N., Matsa R., Lawrenson P., Messenger J., P Walden A. (2015). A UK wide survey on attitudes to point of care ultrasound training amongst clinicians working on the Acute Medical Unit. *Acute Medicine*.

[6] Jahanshir A., Moghari S. M., Ahmadi A., Moghadam P. Z., Bahreini M. (2020). Value of point-of-care ultrasonography compared with computed tomography scan in detecting potential life-threatening conditions in blunt chest trauma patients. *The Ultrasound Journal*.

[7] American College of Emergency Physicians (2001). ACEP emergency ultrasound guidelines-2001. *Annals of Emergency Medicine*.

[8] American College of Emergency Physicians (2009). Emergency ultrasound guidelines. *Annals of Emergency Medicine*.

[9] Legome E., Pancu D. (2004). Future applications for emergency ultrasound. *Emergency Medicine Clinics of North America*.

[10] Whitson M. R., Mayo P. H. (2016). Ultrasonography in the emergency department. *Critical Care*.

[11] Scalea T. M., Rodriguez A., Chiu W. C. (1999). Focused assessment with sonography for trauma (FAST): results from an international consensus conference. *The Journal of Trauma, Injury, Infection, and Critical Care*.

[12] Kirkpatrick A. W., Sirois M., Laupland K. B. (2004). Hand-held thoracic sonography for detecting post-traumatic pneumothoraces: the extended focused assessment with sonography for trauma (EFAST). *The Journal of Trauma, Injury, Infection, and Critical Care*.

[13] Akhtar S., Theodoro D., Gaspari R. (2009). Resident training in emergency ultrasound: consensus recommendations from the 2008 council of emergency medicine residency directors conference. *Academic Emergency Medicine*.

[14] Perera P., Mailhot T., Riley D., Mandavia D. (2010). The RUSH exam: rapid Ultrasound in SHock in the evaluation of the critically lll. *Emergency Medicine Clinics of North America*.

[15] Bekgoz B., Kilicaslan I., Bildik F. (2019). BLUE protocol ultrasonography in Emergency Department patients presenting with acute dyspnea. *The American Journal of Emergency Medicine*.

[16] Manson W., Hafez N. M. (2011). The rapid assessment of dyspnea with ultrasound: RADiUS. *Ultrasound Clinics*.

[17] Jensen M. B., Sloth E., Larsen K. M., Schmidt M. B. (2004). Transthoracic echocardiography for cardiopulmonary monitoring in intensive care. *European Journal of Anaesthesiology*.

[18] Heller T., Wallrauch C., Goblirsch S., Brunetti E. (2012). Focused assessment with sonography for HIV-associated tuberculosis (FASH): a short protocol and a pictorial review. *Critical Ultrasound Journal*.

[19] Léger P., Fleet R., Giguère J. M. (2015). A majority of rural emergency departments in the province of Quebec use point-of-care ultrasound: a cross-sectional survey. *BMC Emergency Medicine*.

[20] Williams J. P., Nathanson R., LoPresti C. M. (2022). Current use, training, and barriers in point-of-care ultrasound in hospital medicine: a national survey of VA hospitals. *Journal of Hospital Medicine*.

[21] Micks T., Sue K., Rogers P. (2016). Barriers to point-of-care ultrasound use in rural emergency departments. *Canadian Journal of Emergency Medicine*.

[22] Sanders J. L., Noble V. E., Raja A. S., Sullivan A. F., Camargo C. A. (2015). Access to and use of point-of-care ultrasound in the emergency department. *Western Journal of Emergency Medicine*.

[23] Khalid B., Aftab A., Kaleelullah S. (2018). Emergency medicine residents’ acquisition of point-of-care ultrasound knowledge and their satisfaction with the flipped classroom andragogy. *POCUS Journal*.

